# Reproducibility of fetal heart volume by 3D-sonography using the XI VOCAL method

**DOI:** 10.1186/1476-7120-8-17

**Published:** 2010-05-12

**Authors:** Enoch Q Barreto, Hérbene F Milani, Edward Araujo Júnior, Karina K Haratz, Liliam C Rolo, Luciano M Nardozza, Hélio A Guimarães Filho, Antonio F Moron

**Affiliations:** 1 Department of Obstetrics, São Paulo Federal University (UNIFESP), São Paulo, SP, Brazil

## Abstract

**Background:**

To assess the reliability of fetal heart volume measurement by three-dimensional sonography (3DUS) using the eXtended Imaging Virtual Organ Computer-aided AnaLysis (XI VOCAL) method.

**Methods:**

This reliability study enrolled 30 pregnant women with singleton healthy pregnancies between 19 and 34 weeks of gestation. All volume acquirements were performed with a convex volumetric transducer (C3-7ED) coupled to an Accuvix XQ sonography device (Medison, Korea). The XI VOCAL 10 planes was the method of choice for volumetric measurement. 3D datasets were analyzed by two observers (EQSB and HJFM); fetal heart volume was measured twice by the first and once by the second observer to calculate intra and interobserver reproducibility. Statistical analysis used pareated Student's t test (p) and calculated Intraclass correlation coefficients (ICC). Bland-Altman plots were also constructed.

**Results:**

We observed an excellent intra- and interobserver reliability for fetal cardiac volume assessed by XI VOCAL. For the intraobserver the ICC was 0.998 (95% CI: 0.997; 0.999), with mean of differences of 0.12 cm^3 ^(95% limits of agreement: -0.84; +0.84; p = 0.130). For interobserver the ICC was 0.899 (95%CI: 0.996; 0.998), mean of differences 0.05 cm^3 ^(95% limits of agreement: -0.84; +0.84; p = 0.175).

**Conclusion:**

Fetal cardiac volume assessed by 3DUS using XI VOCAL method is highly reproducible between 19 to 34 gestational weeks.

## Background

The knowledge of cardiac dimensions *in utero *plays an important role in the assessment of the fetus with congenital heart disease, once these conditions can progress during the prenatal period and lead to severe cardiac decompensation [1,2]. The first cardiac volumetries were performed by two-dimensional (2DUS) sonographic techniques, assuming that the heart presented regularly as an ellipsoid. Three-dimensional sonography (3DUS) came forth in the early 90's providing more accurate volumetric assessment of the heart through the delimitation of its exact external edges [3].

There are classically two methods of volumetric measurement by 3DUS: multiplanar and Virtual Organ Computer-aided AnaLysis (VOCAL). The multiplanar method consists on the display of sequential imaging plans of the object (along its main axis) and the delimitation of the surface of this object in each selected plan. The software processes the area measurements to calculate the final volume of the studied structure [3]. The VOCAL method relies on the rotation of an object around one of its axis, displaying imaging plans that vary in number according to the rotation degree chosen by the operator. The delimitation of the object area is then performed after each rotation. The software ultimately provides the volume value and performs the three-dimensional renderization of the studied object [4]. For the best of our knowledge, there are only two studies in the literature that evaluated fetal cardiac volume by 3DUS, one using multiplanar [5] and the other using the VOCAL method [6]. Those studies proved the good reproducibility of both techniques.

In the latest years a new volumetric technique called eXtended Imaging Virtual Organ Computer-Aided AnaLysis (XI VOCAL) has been available as part of the software pack Three-dimensional eXtended Imaging - 3D XI (Medison, Seoul, Korea). This method relies on the delimitation of sequential adjacent plans presented on the screen (multi-slice view), and the volume calculation is also performed automatically by the computer (adding the areas and multiplying by the distance between them), as is the distance between the initial and last plane studied and the distance between planes [7]. There is only a single study that assessed the fetal volume of fetuses between 11 and 14 weeks *in vivo *using this technique, [8] though there are no descriptions for its use in fetal heart evaluation.

The aim of this study was to assess the intra- and interobserver reliability of fetal heart volumetry by 3DUS using XI VOCAL method.

## Methods

This observational cross-sectional study enrolled 30 pregnant women with singleton healthy pregnancies between 19 and 34 weeks of gestation selected at random. This survey was performed in the 3DUS sector of the Department of Obstetrics of the Federal University of São Paulo (UNIFESP) and was approved by UNIFESP research ethics committee under the register n° 1539/08. All enrolled patients agreed with voluntary participation and signed the informed consent term.

Inclusion criteria were: (1) singleton pregnancy with live fetus; (2) reliable gestational age calculated by the last menstrual period date (LMP) in women with regular menstrual cycles and confirmed by first trimester sonography (based on the CRL measurement). Exclusion criteria were: (1) fetal structural anomalies diagnosed by sonography; (2) patients with chronic conditions that may impair fetal growth; (3) drug abuse or use of prohibited medications during pregnancy; (4) technical difficulty in obtaining the 4 chambers view in the sonographic exam.

All exams were performed in an Accuvix XQ sonography device (Medison, Seoul, Korea), with a convex volumetric multifrequency probe (C3-7ED). All assessments were performed by a single examiner (EQSB) with two years experience in Obstetric 3DUS. Initially a bidimensional live scan was performed in order to access fetal morphology, biometry and estimated weight as well as the amniotic fluid index and placental maturation. The 4 chamber-view plane was used as reference for 3D volume acquisition and as starting plane for volume measurement. 3D data acquisition required fetal absolute rest and maternal short apnea period.

The following parameters were observed: sweep angle varying from 60 to 70° depending on gestational age; highest probe frequency available (5.0 MHz); global gain near to 100%; dynamic range below 80; harmonic mode on. The region of interest (ROI) was adjusted to fit in the whole fetal thorax for the automatic 3D sweep in the fast mode - about 3 seconds. This mode allows the acquisition of higher quality 3D data despite of cardiac movement and frequency of the beats.

**Figure 1:**
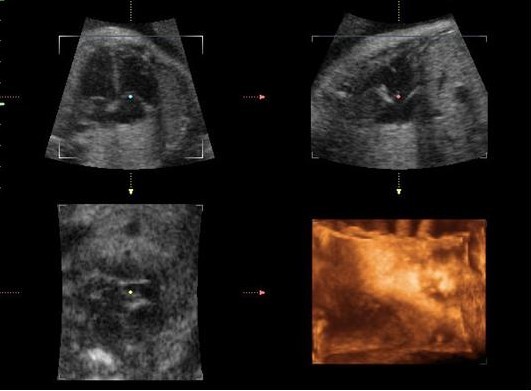
**Image of fetal heart in multiplanar method**. Image of the fetal heart in the three orthogonal planes perpendicular to each other (axial, sagittal and coronal).

3D sets were considered satisfactory when the acquired volume included the whole thorax extension with minimal motion artifacts, otherwise a new scan was performed. After 3D volume scan, the software automatically displayed the image in the multiplanar mode - three orthogonal perpendicular plans (Figure 1). Two 3D sets were acquired for each patient and the best one was chosen for volumetric measurement.

**Figure 2:**
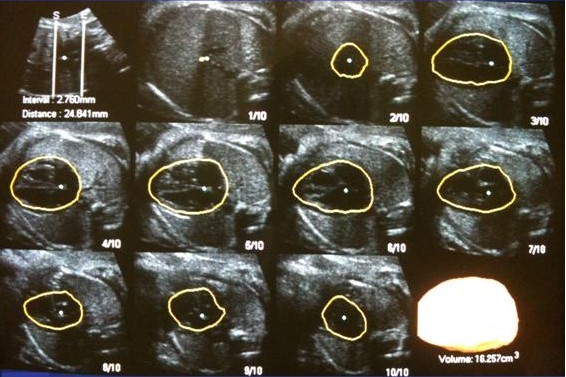
**Fetal heart volume assessment using the XI VOCAL method**. Fetal heart volume assessment using the XI VOCAL method. Axial view with manual outline of 10 sequential planes. XI VOCAL: eXtended Imaging Virtual Organ Computer-Aided AnaLysis.

For the volumetry by XI VOCAL method the first step was setting the initial and ending plans of measurement, according to fetal heart external edges on the reference plane. The mode "manual 10 - planes" was chosen and a diagram of 10 parallel sections of the heart (Multi-slice view - Medison, Seoul, Korea) was displayed on the screen. After manual outline of the external heart surface in all selected planes, the software automatically provides the final cardiac volume, the distance between initial and ending planes, the distance between each section and the 3D renderization of the heart (Figure 2).

The volumetric analysis was performed offline using SonoView Pro 1.03 software (Medison, Seoul, Korea). 3D datasets were analyzed by two observers (EQSB and HJFM); fetal heart volume was measured twice by the first (EQSB) and once by the second observer (HJFM) to calculate intra and interobserver reproducibility. The observers did not have any information about each other's values (blind data). Data were stored in an Excel 2003 (Microsoft Corp., Redmond, WA, USA) worksheet and analyzed by the software Statistical Package for the Social Sciences for Windows version 15.0 (SPSS Inc., Chicago, IL, USA). Reproducibility, (ability of a test to give the same result in different occasions -intraobserver- or between different observers -interobserver variability- was calculated using Student's pareated t-test (p), intraclass correlation coefficient (ICC) and Bland-Altman plots [9]. In Bland-Altman graphical method the differences between the measurements are plotted against the averages of the measurements between two different or the same observer. Horizontal lines are drawn at the mean difference and at the limits of agreement, which are defined as the mean difference plus and minus 1.96 times the standard deviation of the differences. A significance level of 5% was adopted (p < 0.05).

## Results

**Figure 3:**
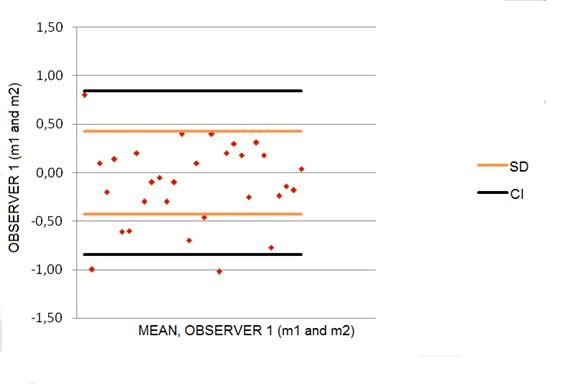
**Differences between measurements performed by a single observer**. Differences between measurements performed by a single observer (m1 and m2) plotted against the mean differences and 1.96 standard deviation. m1: measurement 1; m2: measurement 2.

The population sample was randomly composed of healthy pregnant women of miscegenated Brazilian race, age varying from 17 to 37 years old. 8 patients were primigravidas and 22 multiparous. Only 6 from the 22 multiparous women changed partners in the actual gestation and also 6 had previous cesarean section history. None of the patients had clinical conditions or complications in previous or actual pregnancy. All fetuses were born healthy at term of gestation. The mean maternal age was 24 ± 6.0 years (range 17 - 37 years), the mean gestational age was 26.6 ± 4.85 (range 19 - 34 weeks) and mean parity was 2.43 ± 1.14 (range 1 - 5). For intraobserver reliability paired Student's t-test showed that the two volume measurements made by the same observer to each fetal heart were similar and did not show any statistically significant difference (p = 0.130), the difference being next to zero. ICC shows strong correlation between both measurements (ICC 0.990; 95% CI 0.997; 0.999). Bland-Altman plot demonstrates that 97% of the values were inside the 95% limit of agreement (p < 0.005); the mean of the differences was 0.12 cm^3 ^(95% limits of agreement -0.84; +0.84) with standard deviation of ± 0.44 cm^3 ^(SD) (Figure 3).

**Figure 4:**
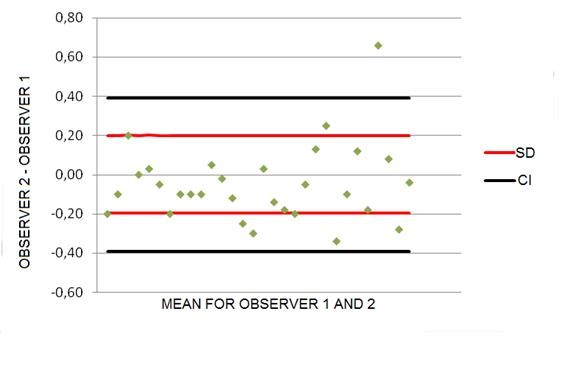
**Differences between measurements performed by two observers**. Differences between measurements performed by two observers (obs1 and obs2) plotted against the mean differences and 1.96 standard deviation. obs1: observer 1; obs2: observer 2.

For interobserver variability paired Student's t-test (p) showed that the assessments made by the two observers were similar and did not show any statistically significant difference (p = 0.175), the difference being next to zero. ICC demonstrated strong correlation between measurements (ICC 0.899; 95% CI 0.996; 0.998). The Bland-Altman plot shows that 97% of the values are inside the 95% limit of agreement (p < 0.005); the mean of the differences was 0.05 cm^3 ^(95% limits of agreement -0.84; +0.84) with standard deviation of ± 0.20 cm^3 ^(Figures 4).

## Discussion

This study proves the good intra- and inter-rater reliability of XI VOCAL method in the assessment of fetal heart volume. There is only one *in vivo *study in the medical literature that compared VOCAL, XI VOCAL and multiplanar techniques in the assessment of the volume of fetuses between 11 and 14 weeks of pregnancy [8]. In this study the authors reported a good intra- and interobserver reproducibility with both techniques (ICC over 0.9 for XI VOCAL 10, 15 and 20 planes).

XI VOCAL technique is a quite recent method. The advantage that this method presents over multiplanar mode is the automatic section of a predetermined number of plans chosen by the operator. It also minimizes imaging artifacts once the 3D dataset doesn't need to be rotated during the volumetry process. Nevertheless, VOCAL XI is still dependent on fetal lie and position, maternal biotype and amount of amniotic fluid and those factors may impair the quality of the three-dimensional data acquired. A recent report validated this technique *in vitro *with high reliability and reproducibility and demonstrated accuracy slightly higher than the rotational technique (VOCAL method) [10].

In our study ICC and the mean of differences were 0.998 and 0.12 cm^3 ^for intraobserver, and 0.899 and 0.05 cm^3 ^for interobserver reproducibility. In a previous study Chang at al [5] assessed fetal cardiac volume of 50 fetuses between 20 to 30 weeks of pregnancy using multiplanar method with 1.0 mm space between sections and found a mean of differences for interobserver reproducibility of -0.03 cm^3^, very similar to our findings. Another study by Peralta et al [6] assessed cardiac volume of 650 fetuses between 12 and 32 weeks of pregnancy using VOCAL method 30° (6 planes) and also observed good intra- and interobserver reproducibility, with mean of the differences of -0.01, -0.02 and 0.15 cm^3 ^for intra- and 0, 0.02 and -0.16 cm^3 ^for interobserver, respectively in gestational weeks intervals of 12-13, 19-22 and 29-32 weeks. The authors do point out commentaries about the standard deviation. The ICC values observed in the present study may in part be related to the relatively regular shape of the fetal heart which makes it easy to be measured once or many times with low variability. We decided to outline 10 planes in the XI VOCAL according to Cheong et al. study [8] that reported an intra and interobserver ICC of 0.995 and 0.942 respectively for this number of sections.

Both VOCAL and multiplanar methods showed to be highly reproducible *in vitro *[4]. However, a survey of fetuses with congenital diaphragmatic hernia (CDH) demonstrated that VOCAL is more accurate when employed in the *in vivo *setting [11]. The lungs of CDH fetuses have very irregular external surfaces and this fact could justify the higher accuracy of VOCAL method in measuring this structures. One of the main advantages of this method compared to multiplanar is that the studied structure outline may be modified in each presented plan during measurement, allowing more precise assessment of irregular objects [6]. Another disadvantage of multiplanar technique is the long time required for each volumetry. Chang et al. reported the duration of 20 to 30 minutes to each heart volume measurement using 1.0 mm space between parallel sections in second/third trimester fetuses [5].

For the best of our knowledge there aren't, until now, any studies that verified XI VOCAL reproducibility *in vitro*, as this method was only recently released [10]. Nevertheless it already presents itself as less prone to transmission artifacts such as acoustic shadows of fetal ribs once it does not need rotation around its axis. This technology, however, is still restricted to only one sonography devices manufacturer.

We believe that this study contribution relies on the presentation of a new technique of measurement fetal cardiac heart volume that is highly reliable. This article opens new perspectives to new studies that intend to determine range values to fetal cardiac volume using XI VOCAL method as others comparing different 3D measurement techniques commercially available. Moreover, XI VOCAL may be used clinically in fetuses at risk for cardiac enlargement due to structural anomalies.

As fetal heart is a relatively regular tridimensional structure, VOCAL method still shows to be more advantageous for its measurement, being faster than multiplanar and more accessible than XI VOCAL. Even though, the latter opens new study perspectives as it has proved its good intra- and interobserver reproducibility in fetal cardiac volumetry.

## Competing interests

The authors declare that they have no competing interests.

## Authors' contributions

EQB: conceived and carried out of the study, collection and analysis of 3D datasets, interview and explanation to patients; HJFM: performed the interobserver variability of the study; EAJ: final review and formatting of the study; KKH: final translation and revision of English; LCR: performed the statistical analysis; LMMN: participated in the design, scientific and methodological review of the study and coordination; HAGF: participated in the scientific and methodological review of the study; AFM: participated in the scientific and methodological review final of the study and coordination.

All authors read and approved the final manuscript.
